# Spatial frequency domain imaging for monitoring immune-mediated chemotherapy treatment response and resistance in a murine breast cancer model

**DOI:** 10.1038/s41598-022-09671-2

**Published:** 2022-04-07

**Authors:** Anup Tank, Cameron Vergato, David J. Waxman, Darren Roblyer

**Affiliations:** 1 Biomedical Engineering, Boston University, Boston, MA USA; 2 Department of Biology and Bioinformatics Program, Boston University, Boston, MA USA

**Keywords:** Optics and photonics, Cancer imaging

## Abstract

Spatial Frequency Domain Imaging (SFDI) can provide longitudinal, label-free, and widefield hemodynamic and scattering measurements of murine tumors in vivo. Our previous work has shown that the reduced scattering coefficient (μ′_s_) at 800 nm, as well as the wavelength dependence of scattering, both have prognostic value in tracking apoptosis and proliferation during treatment with anti-cancer therapies. However, there is limited work in validating these optical biomarkers in clinically relevant tumor models that manifest specific treatment resistance mechanisms that mimic the clinical setting. It was recently demonstrated that metronomic dosing of cyclophosphamide induces a strong anti-tumor immune response and tumor volume reduction in the E0771 murine breast cancer model. This immune activation mechanism can be blocked with an IFNAR-1 antibody, leading to treatment resistance. Here we present a longitudinal study utilizing SFDI to monitor this paired responsive-resistant model for up to 30 days of drug treatment. Mice receiving the immune modulatory metronomic cyclophosphamide schedule had a significant increase in tumor optical scattering compared to mice receiving cyclophosphamide in combination with the IFNAR-1 antibody (9% increase vs 10% decrease on day 5 of treatment, p < 0.001). The magnitude of these differences increased throughout the duration of treatment. Additionally, scattering changes on day 4 of treatment could discriminate responsive versus resistant tumors with an accuracy of 78%, while tumor volume had an accuracy of only 52%. These results validate optical scattering as a promising prognostic biomarker that can discriminate between treatment responsive and resistant tumor models.

## Introduction

The preclinical setting allows for the exploration of new anti-cancer therapies while investigating specific treatment response and resistance mechanisms, providing valuable insight for clinical translation^1^. Preclinical imaging of intra-tumoral functional and metabolic changes induced by chemotherapy and immunotherapy can provide critical information regarding the tumor response and help to identify improved treatment regimens and new methods for clinical treatment monitoring^2^. Recently, a label-free optical imaging technique called Spatial Frequency Domain Imaging (SFDI) has been introduced for preclinical tumor treatment monitoring^3^. SFDI uses spatially modulated near infrared light to provide non-invasive and wide-field measurements of optical absorption and scattering properties^4^. Optical scattering is sensitive to cellular and tissue microarchitecture while absorption is sensitive to tissue concentrations of oxy- and deoxyhemoglobin, which are related to both hypoxia and angiogenesis^5^. SFDI has several distinct advantages over other preclinical imaging modalities. For example, it is uniquely capable of providing non-invasive, contact-free, label-free and widefield optical absorption and scattering maps of tissue. In comparison, microscopy-based intravital imaging methods, such as confocal or multiphoton microscopy, have improved spatial resolution (1–10 μm vs ~500 μm for SFDI) but with limited penetration depth (~ 1 mm vs ~5 mm for SFDI) and often require the addition of contrast agents^6^. Photoacoustic Imaging, which has much deeper penetration (up to several centimeters), is insensitive to scattering changes in tissue^7^. Fluorescence and bioluminescence imaging can provide deep tissue imaging but require exogenous imaging agents or genetically modified animal models, and cannot quantify scattering^8^.

Our group has recently advanced SFDI for preclinical oncology imaging and has established optical scattering as a promising biomarker of drug treatment responses^3, 9–12^. We showed that optical scattering was strongly associated with apoptosis and decreased proliferation induced by cytotoxic and antiangiogenic therapies in murine breast and prostate tumors^3^. Scattering amplitude was positively correlated with cleaved caspase-3, an IHC marker for apoptosis, and increased over time in response to treatment. Additionally, scattering power, which represents the wavelength dependence of scattering, was negatively correlated with PCNA, an IHC marker for proliferation, and decreased over time following treatment. However, an important limitation of this prior work is that the changes in the treated tumors were only compared to untreated controls. This does not adequately reflect the clinical setting in which all patients are treated (i.e., there are no untreated controls). In order to better mimic the clinical setting, models are needed that accurately recapitulate treatment resistance. Examining optical changes in the context of specific resistance mechanisms would then help validate optical scattering as a relevant imaging biomarker and increase the likelihood of translatability to the clinical setting.

To accomplish this, we imaged a paired responsive resistance breast tumor model developed by our group^13^. In this model, metronomic dosing of cyclophosphamide (CPA) in mice bearing E0771 breast tumors induced an immunostimulatory response characterized by infiltration of CD8 + T-cells coupled with a rapid decrease in tumor volume. The treatment response was shown to result from both an immune response and the cytotoxic effects of CPA. This was shown by inhibiting the interferon-1 pathway by blocking interferon-α/β receptor-1 (IFNAR-1) with an antibody during CPA treatment, which greatly inhibited immune cell infiltration, leading to a lack of tumor regression. Type 1 interferon activation has a direct effect on the innate and adaptive response including mediating T-Cell recruitment^14^, which was shown to be required for tumor regression. This is representative of an immunosuppressive model in which CPA would normally successfully treat the tumor, however, the antibody prevented the necessary immune mediated tumor cell death. This paired model of CPA vs CPA + IFNAR-1 antibody identifies immune activation as a key mechanism of response and resistance.

In this work, we used SFDI to image this paired model along with an untreated control tumor group to confirm that optical scattering can serve as a prognostic imaging biomarker to discriminate between responding and resistant tumors. We utilized Generalized Estimating Equations (GEE) to longitudinally model the SFDI data and identify differences in optical parameters between treatment-responsive, treatment-resistant, and control tumors. Additionally, through the use of linear discriminant analysis, the classification accuracy of optical parameters at different timepoints was calculated and compared to the gold standard of tumor volume.

## Methods

### Spatial frequency domain imaging

**Figure 1:**
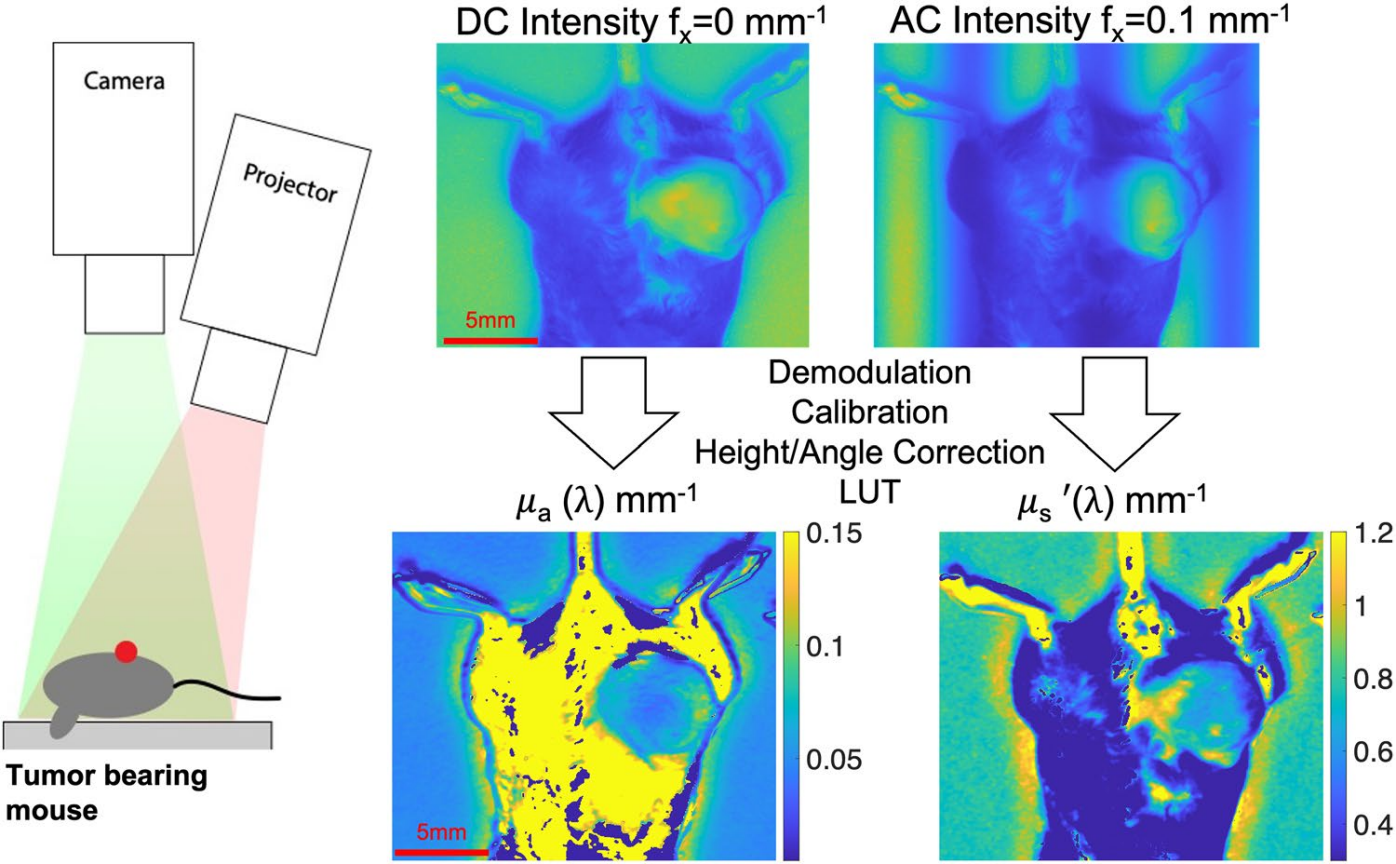
On the left, schematic of the SFDI system with the projector and camera. On the right is an example of intensity images at 731 nm for DC and AC (f_x_ = 0.1 mm^-1^). These are demodulated, calibrated against a phantom, corrected for both height and angle, and then fit to a two-layer lookup table to calculate the optical properties: μ_a_ and μ′_s_.

Specific details about the SFDI methodology, instrumentation and processing have been well described elsewhere^3, 9, 11^. The Reflect RS system (Modulim Inc. Irvine, CA) was utilized to conduct the imaging. Briefly, SFDI was used to extract tissue optical properties: the absorption coefficient (μ_a_) and reduced scattering coefficient (μ′_s_), at each illumination wavelength on a pixel-by-pixel basis shown in Fig. 1.

SFDI projects spatially modulated sinusoidal illumination patterns onto tissue over a 15 × 20 cm field of view. Illumination is provided with LEDs at 659, 691, 731, and 851 nm. For this study, two spatial frequencies, 0 and 0.1 mm^−1^, were projected at each wavelength at 3 different phase offsets (0°, 120°, and 240°) using a digital micromirror device (DMD). A CCD camera was used to collect the remitted light from tissue. Images were demodulated to extract the modulation amplitude envelope for each wavelength-frequency combination using a previously established method^4^. Prior to tissue measurement, a calibration measurement was taken on a tissue mimicking phantom with known optical parameters: (659 nm: μ_a_ =0.0086, μ′_s_ = 1.044; 691 nm: μ_a_ =0.0088, μ′_s_ = 1.014; 731 nm: μ_a_ = 0.0087, μ′_s_ = 0.980; 851 nm: μ_a_ = 0.0074, μ′_s_ = 0.893) to remove the system response and to obtain calibrated diffuse reflectance maps. These measurements were additionally calibrated with the use of an in-frame phantom to correct for any drift that occurs throughout a measurement day, which may span several hours.

The calibrated diffuse reflectance values were then corrected for the height and angle of the tumor^10^. The angle correction algorithm is able to correct for tissues surface angles up to 40-degrees and any angles greater than this threshold were not included in the analysis. The corrected diffuse reflectance values at each pixel were fit to a two-frequency, two-layer lookup table (LUT) to determine the tissue optical properties at each illumination wavelength. This two-layer LUT was developed to account for fixed mouse skin layer optical properties in order to better estimate the underlying tumor optical parameters^9^.

The μ′_s_ at each wavelength was fit to a power law of the form:

$${\mu}_{s}^{{\prime}}(\lambda )=a*{\left(\frac{\lambda }{{\lambda }_{800}}\right)}^{-b}$$

where *a* is the scattering amplitude normalized to 800 nm and *b* is the scattering power. The μ_a_ at each wavelength was fit using Beer’s Law with the known extinction spectra to calculate chromophore concentrations (μM) of oxy-hemoglobin (HbO_2_) and deoxy-hemoglobin (HHb).

$${\mu}_{a}\left(\lambda \right)={\upvarepsilon }_{ {\mathrm{HbO}}_{2}}\left(\lambda \right)*{ct}_{{\mathrm{HbO}}_{2}}+{\upvarepsilon }_{\mathrm{ HHb}}\left(\lambda \right)*{ct}_{\mathrm{HHb}}$$

Additional composite metrics of total hemoglobin (ctTHb = ctHbO_2_ + ctHHb) and oxygen saturation (StO_2_ = ctHbO_2_/ctTHb) were also calculated.

An ROI was manually selected over the tumor region of resulting optical property and hemoglobin maps using the same procedures that have previously been described^3^. Any remaining artifacts in the image were removed such as non-physiological data with extremely low absorption. All data analyzed and displayed represents the means over the ROI. All SFDI processing was conducted using MATLAB R2020b (MathWorks, Natick, Massachusetts).

### Mouse model, treatment, and ex vivo analysis

Five-to six-week-old female C57BL/6 mice (B6-F, Taconic Biosciences, Rensselaer, New York) were implanted orthotopically with 2 × 10^5^ mouse E0771 mammary carcinoma cells in the fourth mammary fat pad. Mice (n = 26) were randomized to three groups: Control, PBS (vehicle) + isotype control (n = 3); CPA, cyclophosphamide (CPA) (Cat # C0768, Sigma-Aldrich, St. Louis, MO) + isotype antibody control (n = 9); and CPA + Ab, CPA + IFNα/β receptor-1 (IFNAR-1) antibody (clone MAR1-5A3, BioXCell, West Lebanon, NH) (n = 14). Treatment was initiated on Day 0 once the tumor volumes reached 250 mm^3^, based on caliper measurements of tumor length (L) and tumor width (W) and the formula: V = (3.14/6) *(L*W)^3/2^. Vehicle control was administered as 200 μL of PBS every 6 days. Isotype control and anti-IFNAR-1 were administered as a 1 mg dose on Day-1, 0.5 mg on Day 0, and 0.25 mg every 3 days afterwards. CPA was administered on a metronomic schedule every 6 days at 110 mg/kg. Volume and SFDI imaging measurements were taken on Days 0–6 and then every 3 days from Days 9 to 30.

Approximately half of the treated mice (CPA, CPA + Ab) were euthanized on Day 12 and the remaining on Day 30 for ex vivo analysis. All the Control mice were euthanized by Day 12. Mice were additionally euthanized when deemed necessary by veterinarian for animal safety and health. Mice were euthanized through CO_2_ asphyxiation and confirmed through cervical dislocation. The exact number of mice in each treatment category at each time point are shown in Figure S1. All animal work was reviewed and approved by the Boston University Institutional Animal Care and Use Committee. All animal experiments were performed in accordance with the necessary and relevant guidelines. This manuscript follows the reporting recommendations of the ARRIVE guidelines.

### Statistical analysis

Generalized estimating equations (GEE) were used to longitudinally model the SFDI optical parameters and tumor volume throughout the course of the study using SAS (SAS institute)^15^. GEEs are a method to model the population averaged effects for longitudinal data with repeated measurements. All parameters were normalized to baseline values at day 0 and presented as a percent change from baseline in order to normalize inter-mouse differences. The GEE incorporates the information from repeated measurements from an individual to better model the population means. The GEE allowed for an unbalanced longitudinal dataset with subjects considered as clusters, an autoregressive correlation structure, and a normal model with identity link function. Separate models were run for the outcome variables of interest: scattering amplitude (*a*), deoxy-hemoglobin concentration (ctHHb), and tumor volume. Separate models were run on the short-term trends consisting of all three treatment groups until Day 12, when there are at least 3 mice per treatment category and the long-term trends, consisting of the CPA and CPA + Ab group until Day 30. Significance between the model parameters was determined at 0.050 and when adjusted for multiple comparisons at 0.017. Post-hoc contrasts for outcome variables adjusted for model covariates were compared across treatment groups at each measurement day. Significance for the post-hoc contrasts was determined at 0.050 and when adjusted for multiple comparisons was at 0.007 for the short-term analysis and 0.003 for the long-term analysis.

Linear discriminant analysis (LDA) was performed to assess the classification accuracy of the SFDI-derived optical parameters to discriminate the different treatment groups relative to the reference of tumor volume. The LDA assumed multivariate normal distributions and equal covariances for each group. The analysis utilized leave-one-out cross validation to limit biasing the classifier. Similar to the GEE analysis, two separate analyses were conducted: (1) a short-term analysis consisting of all treatment groups up to Day 6 and (2) a long-term analysis consisting of CPA and CPA + Ab treatment groups up to Day 30. Due to the presence of three groups, the figure of merit was classification accuracy ((Correctly Classified Cases)/All Cases).

## Results

### Regimen specific volume changes

**Figure 2:**
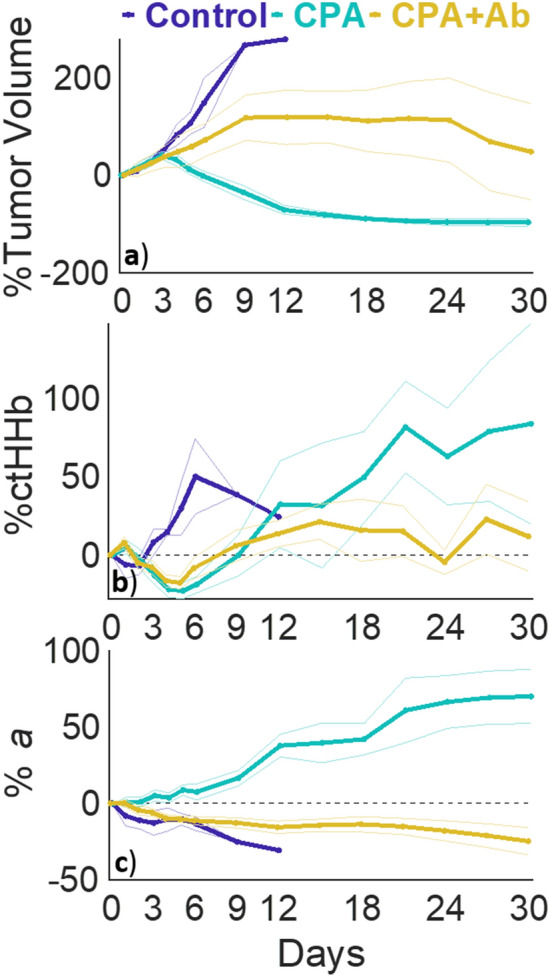
Longitudinal day 30 volume and optical changes across treatment groups. Percent change in (**a**) tumor volume, (**b**) ctHHb, (**c**) *a* separated by treatment: Control (purple), CPA (blue), CPA + Antibody (yellow). Lines represent means and shaded bars represent standard errors.

Tumor volume changes are shown in Fig. 2a with day 0 representing the first day of treatment when tumor volume reached 250 mm^3^, between 21–24 days after inoculation. The CPA group showed an increase in volume until day 3 when the tumor volume began to sharply decrease. The Control and CPA + Ab groups had large increases in tumor volume. Control group tumors appeared to undergo exponential growth, and all mice in this group were euthanized on Day 12. The CPA + Ab group showed a linear increase in tumor volume until approximately Day 9, followed by growth stasis.

### Regimen specific ex vivo analysis

The ex vivo analysis is extensively described in the companion publication^13^. Briefly, using qPCR and fluorescence activated cell sorting, it was determined that there was a transient upregulation of immune stimulated genes such as MX1 and a subsequent infiltration of CD8 + T-Cells in the CPA group. This is in contrast to the CPA + Ab group, in which there was limited upregulation of immune stimulated genes and a lack of infiltrating CD8 + T-cells.

### Regimen specific optical changes

**Figure 3:**
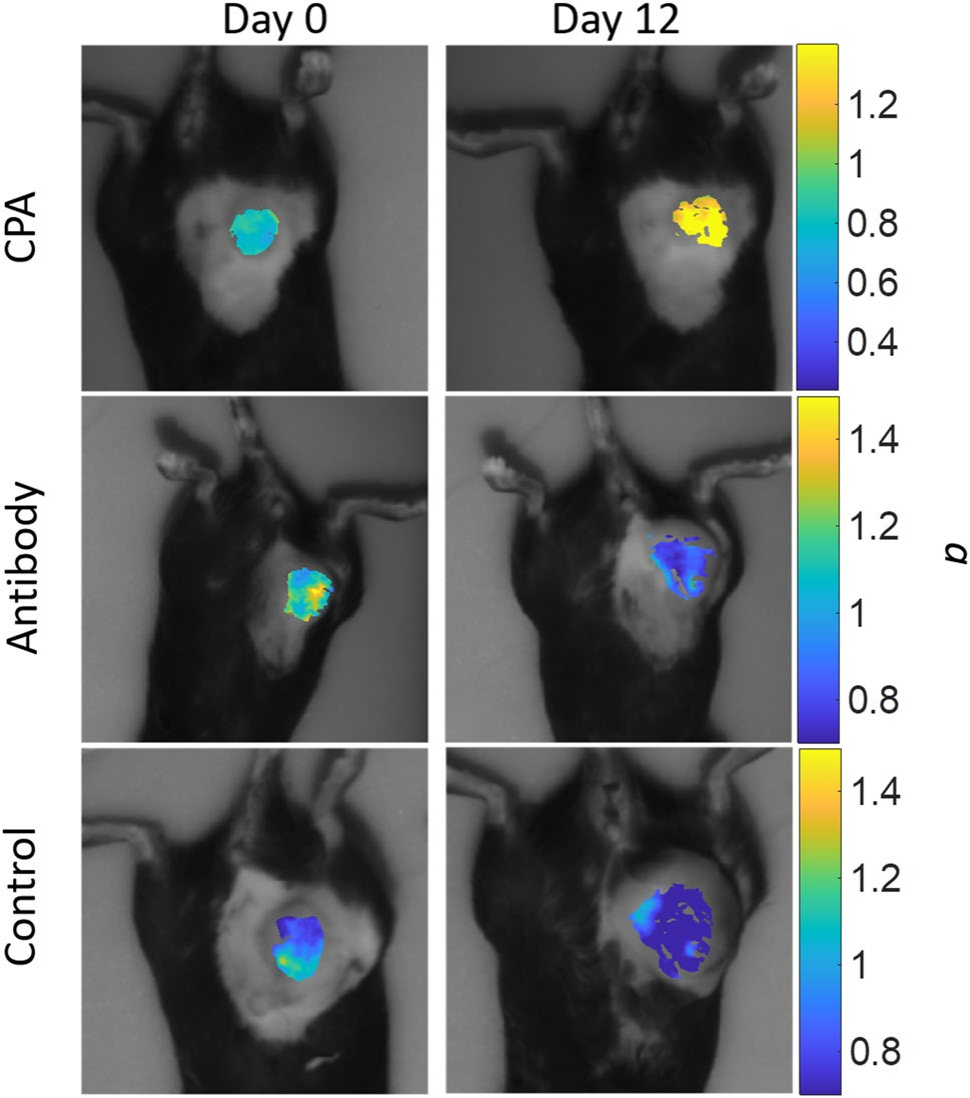
SFDI maps across treatment groups. Representative wide-field SFDI maps of the changes in *a* parameter (mm^−1^) at Day 0 and 12 across the each of the three treatment groups.

SFDI optical changes were differentially modified by their specific treatment. Two parameters, ctHHb and *a*, manifested markedly different patterns based on treatment, as shown in Fig. 2b,c, respectively. ctHHb increased dramatically in the Control group over the early measurement period (Days 3–6) compared to both treated groups, which both showed a small decrease in the same time period. The scattering amplitude, *a*, increased dramatically over time in the CPA treatment group, while the Control and CPA + Ab groups both showed small decreases throughout their respective treatment course. The other longitudinal optical parameters measured by SFDI are shown in Supplemental Figs. 2–5. Representative widefield *a* maps for each treatment group are shown in Fig. 3. The differences between the CPA and other treatment groups increased over time for both ctHHb and *a*.

### Longitudinal analysis

GEE Analysis demonstrated that Tumor Volume, ctHHb and *a* all had significant covariates of time * treatment group. Post hoc Analysis of the comparisons between the different treatment groups at different time points are shown visualized in Supplementary Tables 1–3. Volume was significantly different between the CPA and Control group starting on Day 3 and the magnitude of the change increased each subsequent day. The CPA + Ab group was significantly different from the CPA and Control group starting on Day 5 and the difference between the groups continued to increase each subsequent day. ctHHb was not significantly different between the CPA + Ab and CPA group at any time point. ctHHb was significantly different between the Control and both treatment groups during Days 4–6. After Day 6, the changes between the groups started to decrease. *a* was significantly different from the CPA group relative to the other groups starting on Day 5, and the differences tended to increase at subsequent time points.

### Discriminant analysis

**Figure 4:**
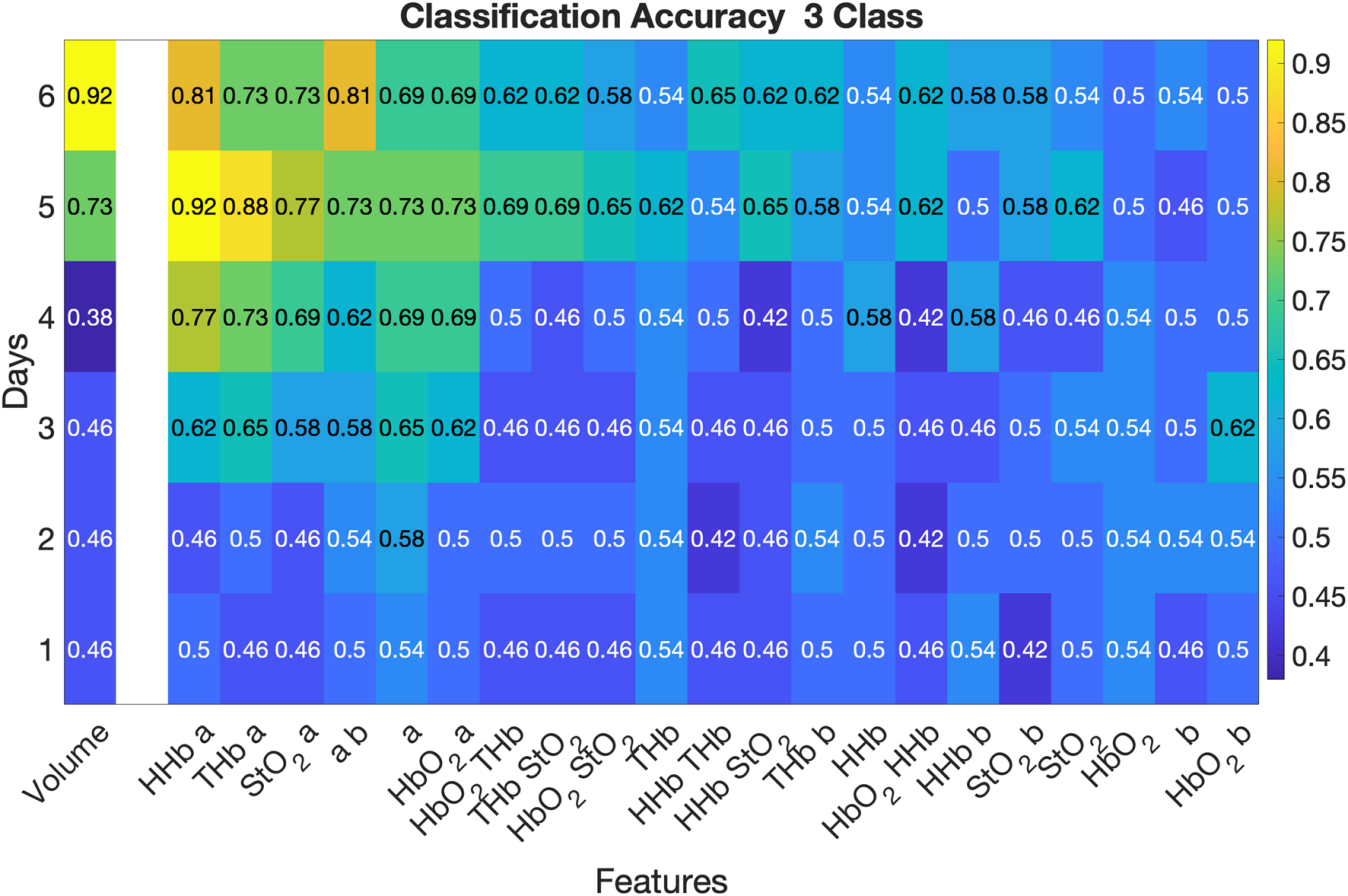
Short term classification accuracy feature selection. Linear discriminant analysis was conducted on the short-term cohort (through Day 6) for all 3 treatment groups (Control, CPA, and CPA + Ab). The classification accuracy for each timepoint and feature is displayed in text and indicated by shading for each metric at each timepoint. The combination of the SFDI parameters ctHHb and *a* provided higher classification accuracy than tumor volume on days 3, 4, and 5.

Linear discriminant analysis demonstrated that in the short-term analysis the best combination of optical features was ctHHb and *a* on Day 5 with an accuracy of 0.92 to discriminate between the three treatment groups, as shown in Fig. 4. This was only matched by the tumor volume metric on Day 6 with an accuracy of 0.92. Additionally, on Days 3, 4, and 5 the ctHHb and *a* metric had a substantially larger accuracy compared to the single feature of volume. The best single optical feature was *a*, which was equivalent to or outperformed volume on Days 3, 4, and 5.

**Figure 5:**
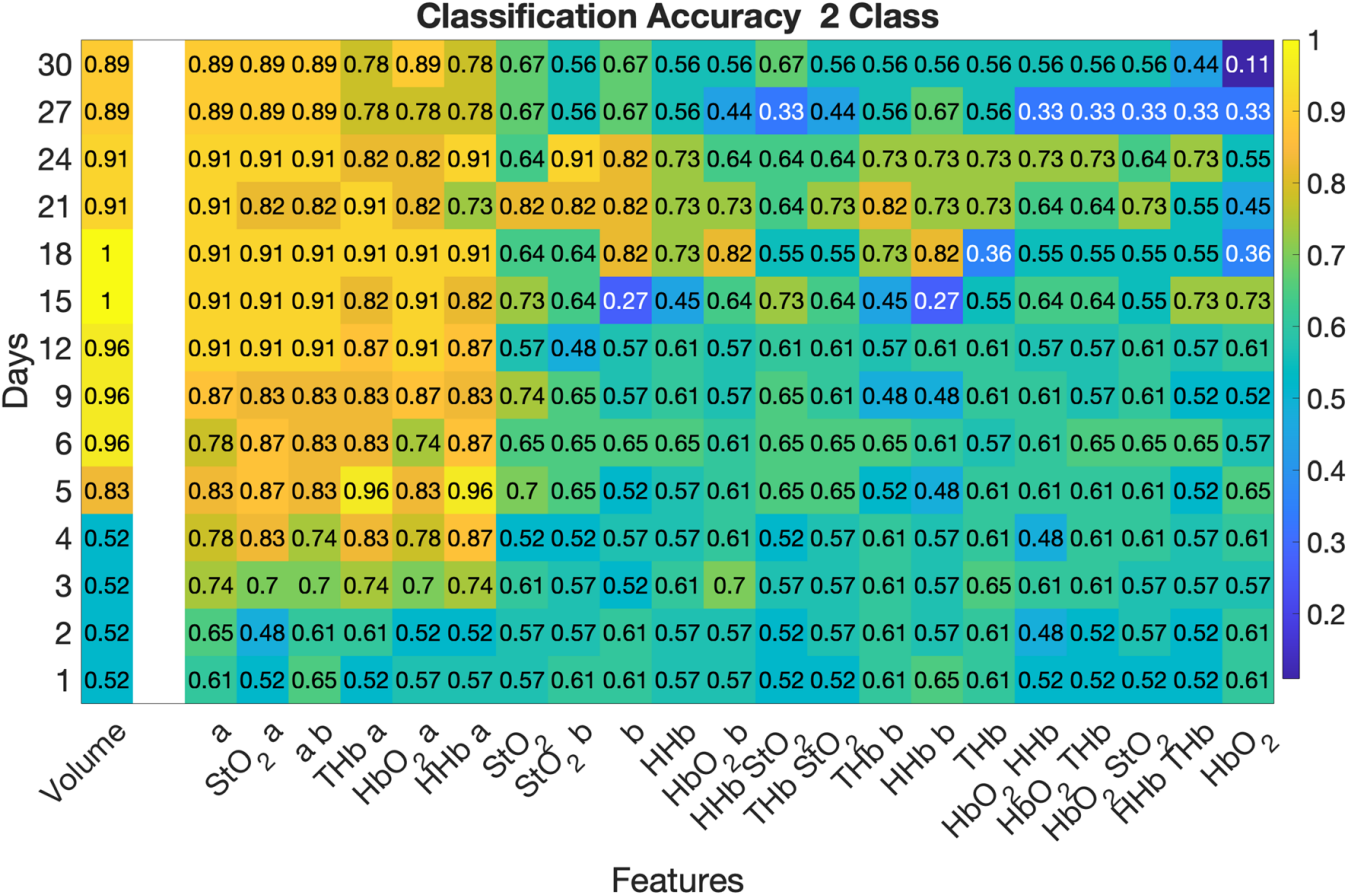
Long term classification accuracy feature selection for responders (CPA) versus resistant (CPA + Ab) cohorts. Linear discriminant analysis was conducted on the long-term cohort (through Day 30) for the treatment groups. The classification accuracy for each time point and feature is displayed in text and indicated by shading for each metric at each time point. SFDI derived parameters predicted earlier ability to discriminate responders versus resistance mice compared to tumor volume.

**Figure 6:**
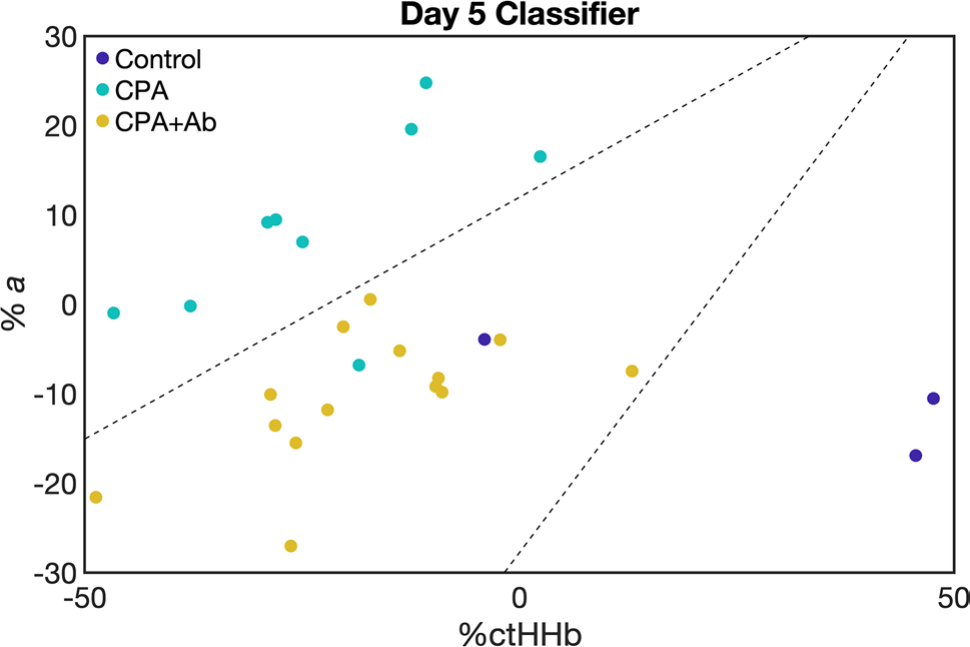
2 Feature Classification at Day 5. Scatterplot of the 2 best classification features: ctHHb and *a* for individual mice. The color of the individual points indicates its respective treatment: Control (purple), CPA (blue), CPA + Ab (yellow). The dashed lines indicate the calculated lines of separation between the different treatment groups.

For the long-term analysis shown in Fig. 5, *a* as a single feature was the best optical discriminator between the CPA and the CPA + Ab group over the entire study. It was a superior discriminator compared to tumor volume for the first five days, after which volume either matched or exceeded classification accuracy for the duration of the study. The individual trends in ctHHb and *a* along with tumor volume on Day 5 are shown in Supplemental Fig. 6. These data, color coded by treatment, visually demonstrate how each treatment group displays a distinct trend. Figure 6 presents a scatterplot of the relationship between ctHHb and *a* for individual tumors on Day 5, with dashed lines indicating the lines of separation between each group. This figure shows that with an overall accuracy of 0.92, there were 2 misclassifications out of 24 subjects: one of each of the CPA and Control groups were mistakenly determined to be in CPA + Ab group.

## Discussion and conclusion

This works demonstrates the utility of SFDI to identify novel prognostic optical biomarkers for immune-modulated chemotherapy response and resistance. Specifically, scattering amplitude, *a*, was shown to be capable of accurately discriminating between a paired treatment-responsive and treatment-resistant murine breast cancer model at early time points. Dramatic changes in *a* occurred prior to changes in tumor volume, demonstrating its potential prognostic value as an early predictor of tumor response and resistance.

The two treatment groups and one control group exhibited dramatically different longitudinal tumor volume trends. The untreated control group showed a large, exponential increase in tumor volume, as expected. The CPA + Ab group displayed a steady linear increase in tumor volume until Day 12, when it reached tumor stasis, likely representing an immunosuppressive phenotype. The CPA group showed a steady decrease in tumor volume after Day 3 and until Day 12, demonstrating a treatment-responsive and likely immunostimulatory phenotype. These results demonstrate a paired model of treatment response and resistance directly linked to immune stimulation or inhibition, respectively.

Starting on Day 5, the *a* parameter showed significant differences through GEE analysis (p < 0.005) when comparing the CPA group (9% increase) to both the CPA + Ab (10% decrease) and Control (10% decrease) groups. This difference continued to grow throughout the study. This finding builds upon the previous findings by our lab that *a* can serve as a prognostic biomarker of treatment response in both prostate and breast cancer models^3^.

The *a* parameter also showed predictive ability to discriminate between responsive, resistant, and untreated control tumors using linear discriminant analysis. The combination of ctHHb and *a* had high predictive ability to separate the three groups and outperformed tumor volume on Days 3, 4, and 5. When comparing the responsive CPA and resistant CPA + Ab, only one CPA mouse was misclassified. This means that all of the resistant tumors were accurately identified, which may represent the most important use of this imaging biomarker. The *a* parameter was equal to or outperformed tumor volume as a classification feature on the first 5 days of study when discriminating between the CPA and the CPA + Ab group, and demonstrated excellent classification accuracy (~ 0.9) throughout the rest of the study. We note that the optical parameters used in the analysis were normalized to their pretreatment values and were represented as percent changes from baseline. This may help to improve the translatability of these findings as the classification accuracy relied only on relative changes rather than absolute tumor optical properties, which are likely to be highly variable in a diverse clinical population.

The changes observed in the *a* parameter are consistent with our prior work, which demonstrated that increases in the *a* parameter were associated with increased apoptosis as determined by ex-vivo immunostaining^3^. Apoptosis induces a dramatic shift in the microarchitecture of a cell^16^. The chromatin in the nucleus deforms into aggregates before the cell nucleus breaks apart into smaller pieces. This in turn increases the local density of scattering centers which has been shown to increase the *a* parameter^17, 18^.

We also note that in the companion paper describing the model used here, the CPA treated groups tended to show cytotoxic CD8 + T-Cell infiltration by Day 6, (see Fig. 5 of Ref.^13^), which is approximately when the scattering changes between the groups began to differentiate. This suggests that T-Cells contribute to treatment response as cytotoxic CD8 + T-cells most commonly kill cancer cells through apoptotic pathways^19^. Clinically, the presence of tumor infiltrating lymphocytes and specifically CD8 + T-cells has been associated with improved outcomes^20, 21^. It has previously been shown that the infiltration of CD8 + T-cells in EO771 tumors receiving immune checkpoint blockade therapy was necessary for tumor response, and was associated with improved vessel perfusion^22^. Interestingly, in this work, CPA treatment was associated with an increase in ctHHb, ctTHb and a drop in StO_2_ after day 6 (Figs. 2b and Supplementary Figures S3 and S4), suggesting a higher tumor blood volume and a more hypoxic phenotype.

While the study focused on optical scattering changes, it is of note that the changes in ctHHb in the control group were significantly different from the treatment groups between Days 3 and 6. The large early spike in ctHHb in the control group may be correlated to the rapid tumor growth during this time period, potentially indicating high metabolic activity, and the tumor outgrowing its vascular supply^23^. Interestingly, after Day 9, ctHHb of the CPA group continued to increase through the end of the study, despite the fact that the tumor volume decreased or reached stasis. This is in contrast to the CPA + Ab group that approximately followed the same trends as the CPA group until Day 15, after which ctHHb started to decrease and then stabilized around a smaller value (25% vs 50%). This could potentially be due to the fact that the tumor volume of the CPA + Ab group stabilized at this point and was no longer rapidly growing.

There are several limitations to this study. First, this study did not assess the optical properties in healthy tissue such as the contralateral fat pad, which may have changed with treatment. Second, this study did not compare the tumoral effect of administering the IFNAR-1 antibody in the absence of CPA. The IFNAR-1 antibody blocks a major immune pathway, which could have downstream effects on tumor growth and other functional changes. We note however that in a prior study in a mouse glioma model that IFNAR-1 antibody had no effect on tumor growth in the absence of CPA treatment^24^. Here its use was solely to block the immune stimulation caused by metronomic CPA^13^. Third, a limited number of untreated control mice (n = 3) were utilized. This was because the primary aim was to compare the changes between groups receiving treatment. Fourth, SFDI has a relatively shallow penetration depth (~ 5 mm) and may not be sensitive to changes in deeper tissue. Fifth depth sectioning was not explored with SFDI here, potentially obfuscating spatial heterogeneity such as differences in the core compared to the periphery of the tumor. Finally, while SFDI has been extensively used in various clinical applications including breast cancer treatment monitoring, its limited depth sensitivity could be a significant challenge in clinical setting with deeper tumors^25, 26^. Instead, other diffuse optical imaging technologies such as frequency domain- or time domain-diffuse optical spectroscopy may be better suited for tracking the scattering changes in tumors^27^. These clinical technologies allow for the potential for translation and validation of biomarkers found in the preclinical setting with SFDI. There has been limited clinical work in examining scattering as a prognostic biomarker with diffuse optics, representing a potential avenue for future study.

Identification of prognostic biomarkers for treatment response remains a critical factor for improving treatment response. We have demonstrated SFDI derived optical scattering can serve as a promising prognostic marker to differentiate immune response and ultimately tumor response. This validates SFDI as a tool to investigate tumoral functional and metabolic changes when exposed to agents with varying mechanisms of actions such as immunotherapies. Importantly, this also raises the potential of using SFDI to discover predictive markers of resistance to treatment.

## Supplementary Information

Supplementary Information.

## Data Availability

The datasets generated during and/or analyzed during the current study are available from the corresponding author on reasonable request.
