# Supplementary material for: Spatial frequency domain imaging for monitoring immune-mediated chemotherapy treatment response and resistance in a murine breast cancer model
Source: Sci Rep. 2022 Apr 7;12:5864. doi: 10.1038/s41598-022-09671-2 (PMC8989878; doi:10.1038/s41598-022-09671-2)
Supplement: Supplementary file 1 — Supplementary Information. [file 41598_2022_9671_MOESM1_ESM.docx]

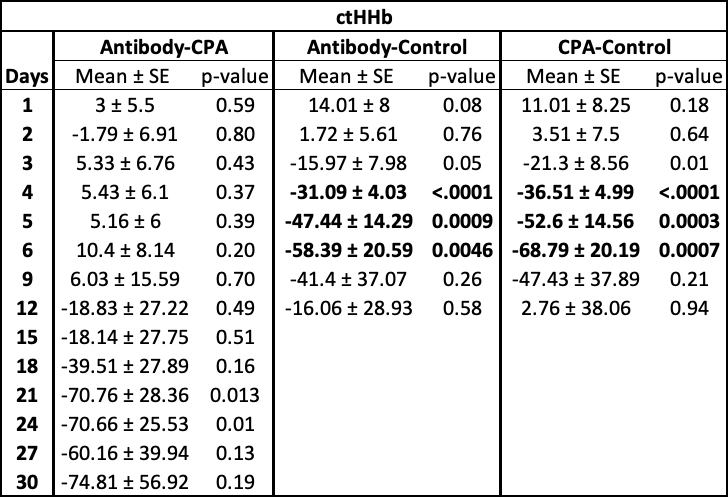


**Supplementary Table 1**

Post hoc GEE contrast for ctHHb treatment comparisons

Post hoc GEE contrasts for ctHHb adjusted for model covariates comparing each pair of treatment groups at each treatment day with the mean and standard error along with the p-value. Significance for the short term analysis was determined at 0.007 and long term analysis at 0.003 and is indicated by bolding.


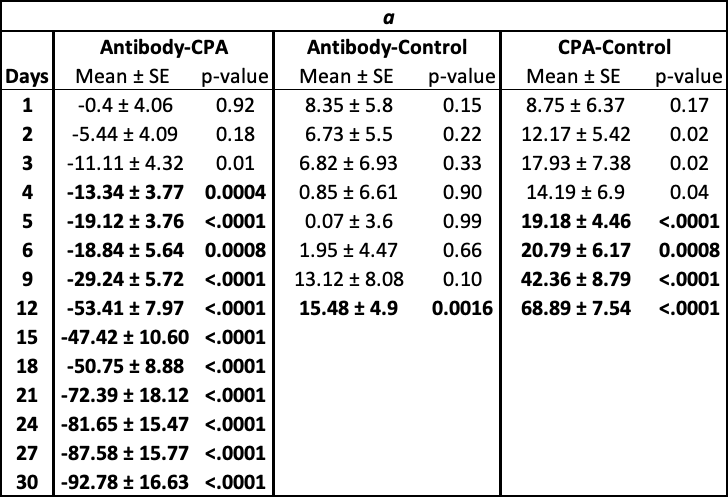


**Supplementary Table 2**

Post hoc GEE contrast for *a* treatment comparisons

Post hoc GEE contrasts for *a* adjusted for model covariates comparing each pair of treatment groups at each treatment day with the mean and standard error along with the p-value. Significance for the short term analysis was determined at 0.007 and long term analysis at 0.003 and is indicated by bolding.


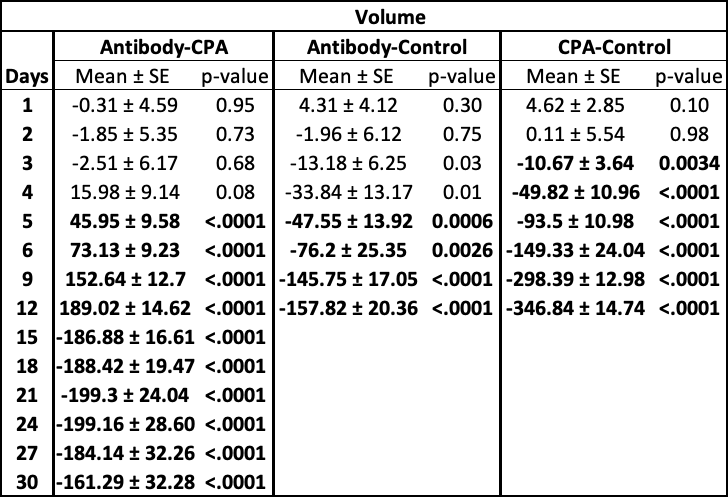


**Supplementary Table 3**

Post hoc GEE contrast for volume treatment comparisons

Post hoc GEE contrasts for volume adjusted for model covariates comparing each pair of treatment groups at each treatment day with the mean and standard error along with the p-value. Significance for the short term analysis was determined at 0.007 and long term analysis at 0.003 and is indicated by bolding.

**Supplementary Figure 1**

Bar graph displaying the number of mice measured with each SFDI at each timepoint separated by each treatment group: Control (purple), CPA (blue), CPA + Antibody (yellow).


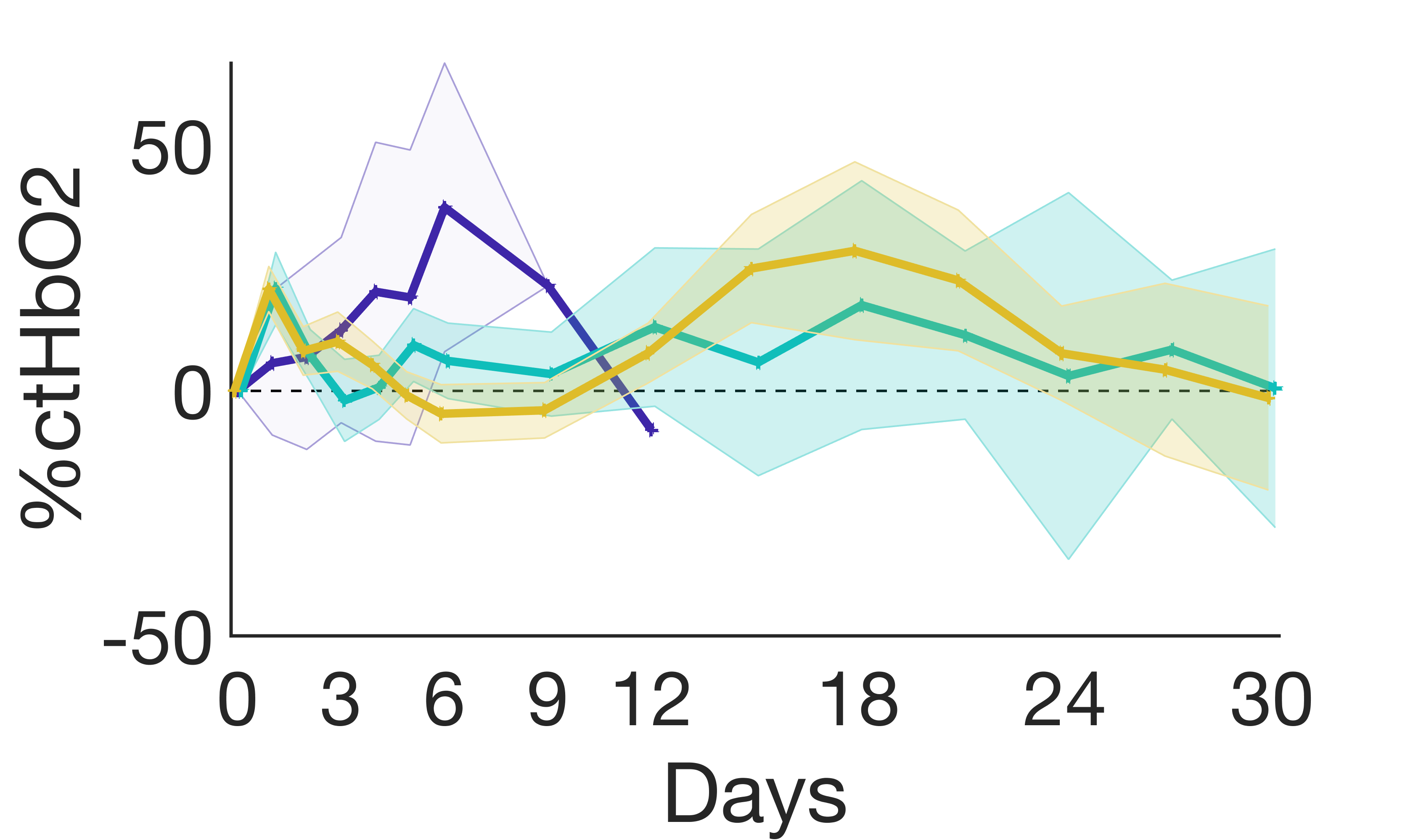


**Supplementary Figure 2**

Longitudinal Day 30 percent change in ctHbO2 Across Treatment Groups. Percent change in ctHbO2 separated by treatment: Control (purple), CPA (blue), CPA + Antibody (yellow). Lines represent means and shaded bars represent standard errors.


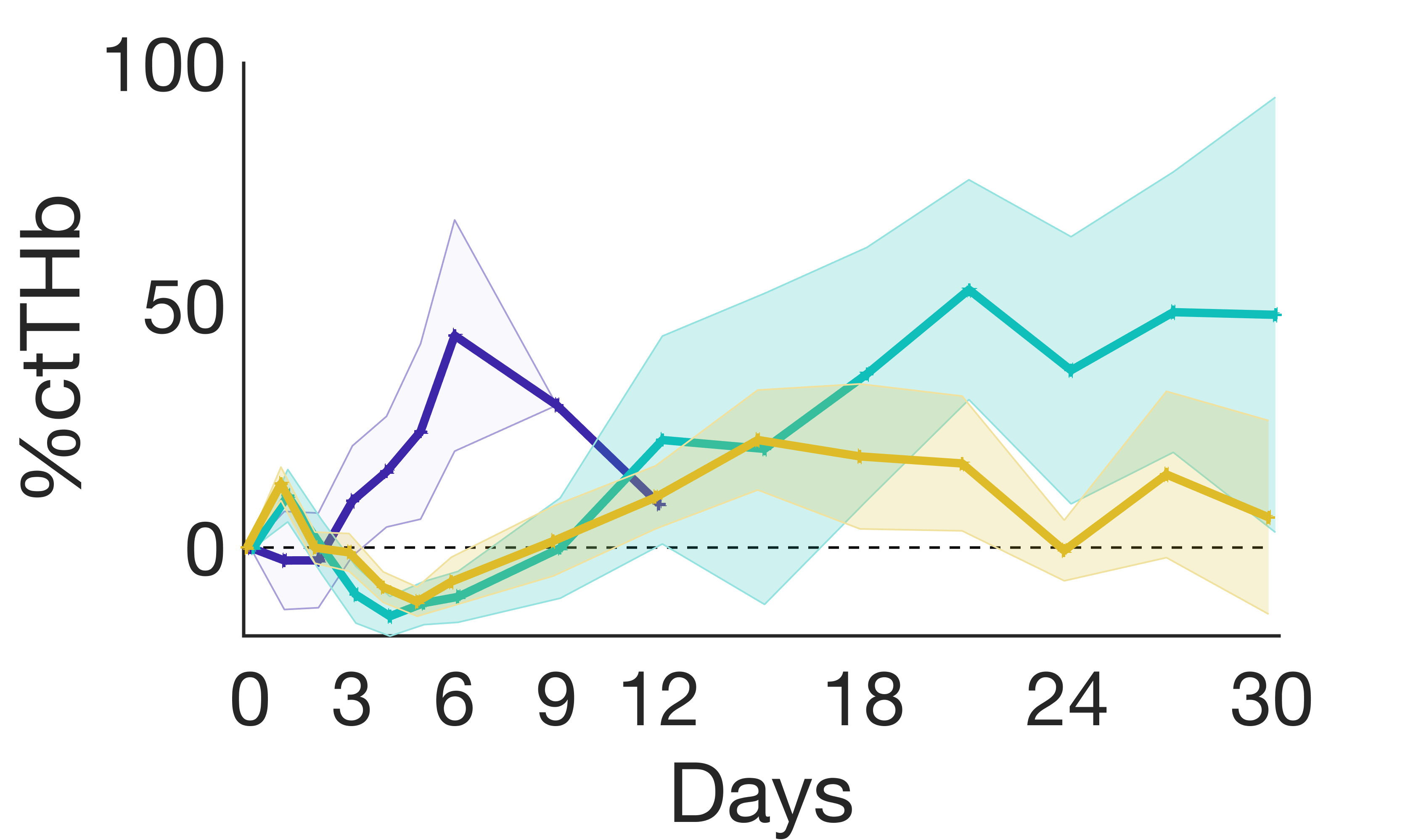


**Supplementary Figure 3**

Longitudinal Day 30 percent change in ctTHb Across Treatment Groups. Percent change in ctHbO2 separated by treatment: Control (purple), CPA (blue), CPA + Antibody (yellow). Lines represent means and shaded bars represent standard errors.


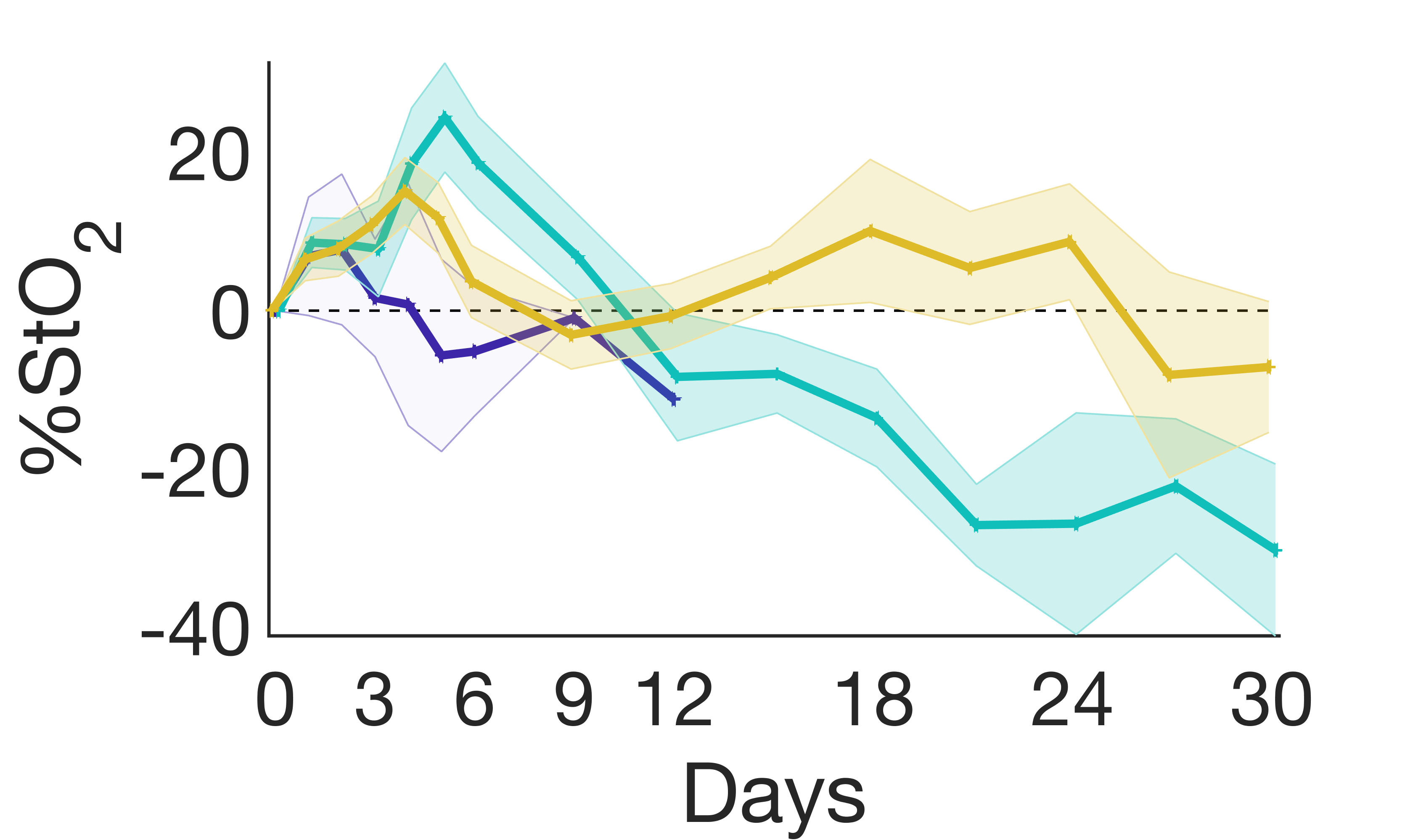


**Supplementary Figure 4**

Longitudinal Day 30 percent change in stO_2_ Across Treatment Groups. Percent change in ctHbO2 separated by treatment: Control (purple), CPA (blue), CPA + Antibody (yellow). Lines represent means and shaded bars represent standard errors.


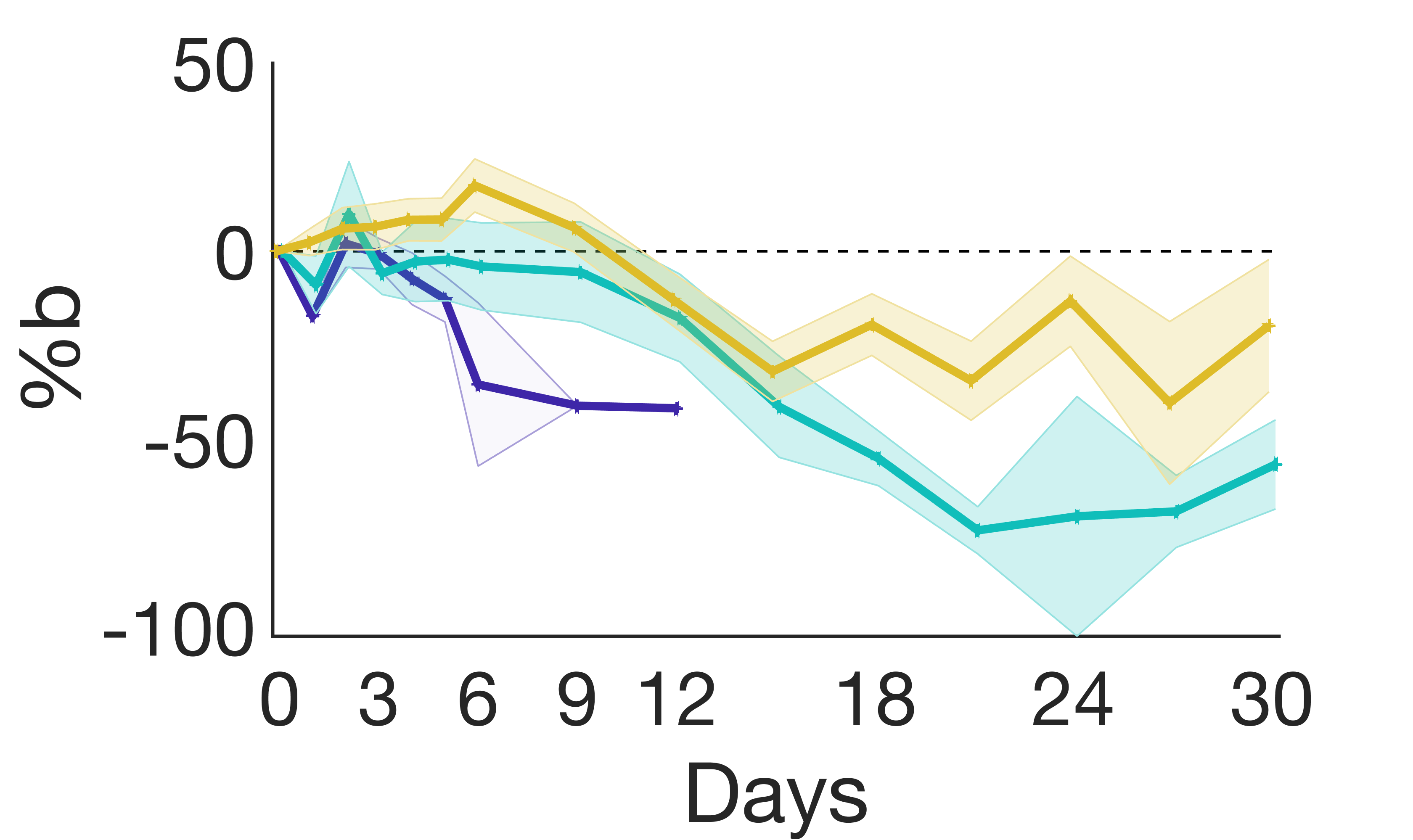


**Supplementary Figure 5**

Longitudinal Day 30 percent change in b Across Treatment Groups. Percent change in ctHbO2 separated by treatment: Control (purple), CPA (blue), CPA + Antibody (yellow). Lines represent means and shaded bars represent standard errors.


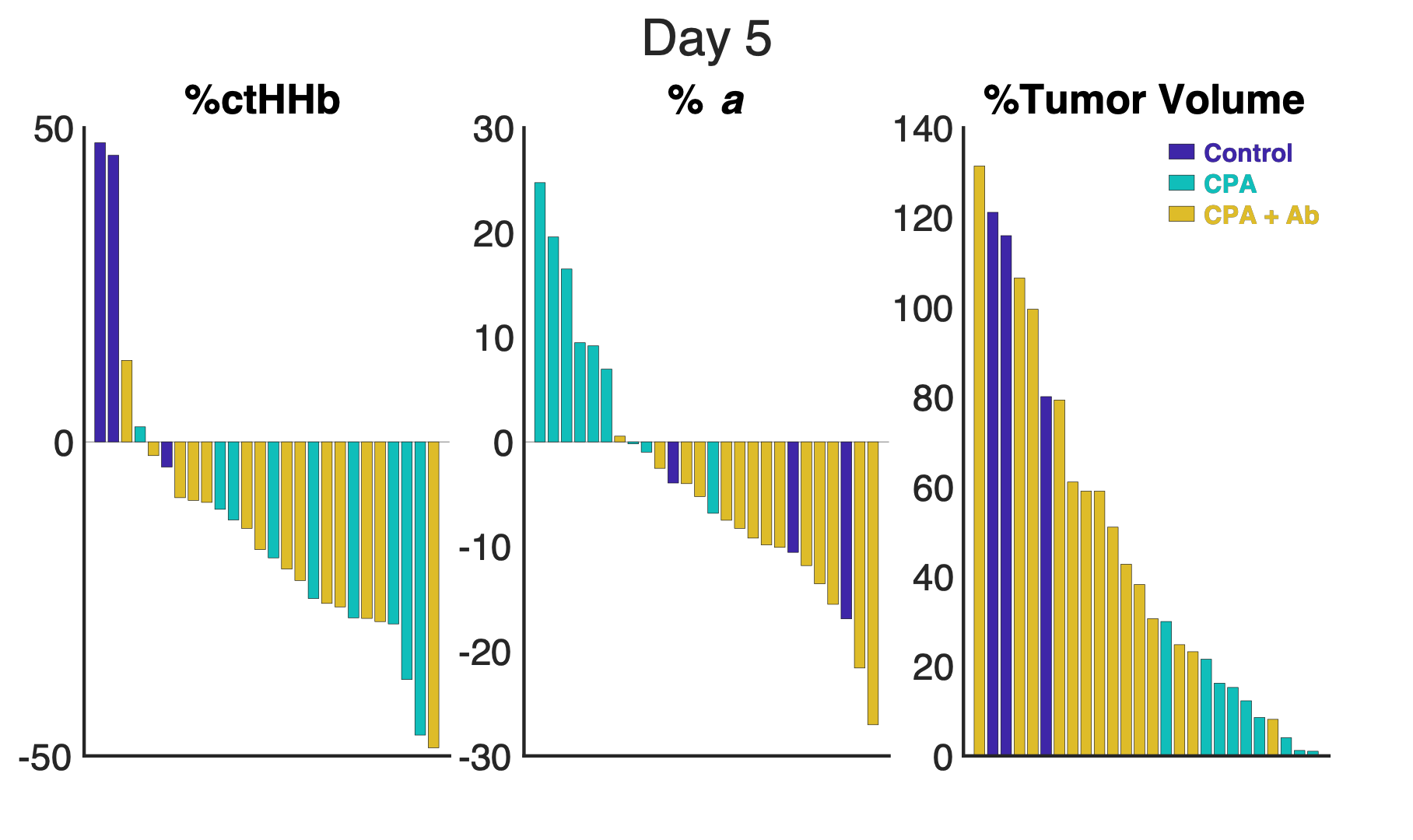


**Supplemental Figure 6**

Waterfall Plot of Individual mice optical and volume changes on Day 5. Plots of individual mice changes in ctHHb, *a*, and tumor volume on Day 5. The color of the bar plot indicates its respective treatment: Control (purple), CPA (blue), CPA + Antibody (yellow)
